# Parkinson’s disease multimodal complex treatment improves gait performance: an exploratory wearable digital device-supported study

**DOI:** 10.1007/s00415-022-11257-x

**Published:** 2022-07-21

**Authors:** Raphael Scherbaum, Andreas Moewius, Judith Oppermann, Johanna Geritz, Clint Hansen, Ralf Gold, Walter Maetzler, Lars Tönges

**Affiliations:** 1 Department of Neurology, St. Josef-Hospital, Ruhr University Bochum, 44791 Bochum, Germany; 2 Department of Neurology, Christian-Albrechts-University of Kiel, Kiel, Germany; 3 Neurodegeneration Research, Protein Research Unit Ruhr (PURE), Ruhr University Bochum, 44801 Bochum, Germany

**Keywords:** Parkinson’s disease, Multidisciplinary, Inpatient, Wearable digital devices, Gait, Balance

## Abstract

**Background:**

Wearable device-based parameters (DBP) objectively describe gait and balance impairment in Parkinson’s disease (PD). We sought to investigate correlations between DBP of gait and balance and clinical scores, their respective changes throughout the inpatient multidisciplinary Parkinson’s Disease Multimodal Complex Treatment (PD-MCT), and correlations between their changes.

**Methods:**

This exploratory observational study assessed 10 DBP and clinical scores at the start (T1) and end (T2) of a two-week PD-MCT of 25 PD in patients (mean age: 66.9 years, median HY stage: 2.5). Subjects performed four straight walking tasks under single- and dual-task conditions, and four balance tasks.

**Results:**

At T1, reduced gait velocity and larger sway area correlated with motor severity. Shorter strides during motor-motor dual-tasking correlated with motor complications. From T1 to T2, gait velocity improved, especially under dual-task conditions, stride length increased for motor-motor dual-tasking, and clinical scores measuring motor severity, balance, dexterity, executive functions, and motor complications changed favorably. Other gait parameters did not change significantly. Changes in motor complications, motor severity, and fear of falling correlated with changes in stride length, sway area, and measures of gait stability, respectively.

**Conclusion:**

DBP of gait and balance reflect clinical scores, e.g., those of motor severity. PD-MCT significantly improves gait velocity and stride length and favorably affects additional DBP. Motor complications and fear of falling are factors that may influence the response to PD-MCT. A DBP-based assessment on admission to PD inpatient treatment could allow for more individualized therapy that can improve outcomes.

**Trial registration number and date:**

DRKS00020948 number, 30-Mar-2020, retrospectively registered.

**Supplementary Information:**

The online version contains supplementary material available at 10.1007/s00415-022-11257-x.

## Introduction

Parkinson’s disease (PD) impairs patients with both non-motor [1] and motor symptoms such as bradykinesia, rigidity, tremor, and postural instability [2]. Among the most disabling PD symptoms are gait and balance impairments [3] which are both progressive during the course of the disease [4].

Mobility limitations due to gait and balance impairment substantially contribute to the poor quality of life of people living with PD [5–8]. This effect is likely mediated by the negative impact of gait and balance impairment on daily function [9, 10] which is the main determinant of quality of life [11]. Accordingly, independent walking is perceived as a precondition for autonomy and participation in society by people with PD [12].

To enhance the quality of life in patients with advanced PD, a multidisciplinary approach is considered to be beneficial [13, 14]. In such multidisciplinary team approaches, traditional pharmacological treatment is complemented by non-pharmacological therapies such as physiotherapy [15], occupational therapy, and speech and language therapy. Thereby, partially insufficient effects of dopaminergic therapy, e.g., on axial motor functions [16] are counterbalanced by positive effects of exercise on gait and balance [17]. The Parkinson’s Disease Multimodal Complex Treatment (PD-MCT) is a multidisciplinary inpatient approach with favorable effects on motor symptoms and quality of life [18–22] which is applied up to 24% of all PD inpatients in Germany [20]. It is guided by the principles of individualized [23], i.e., tailored, and person-centered [24] health care.

Response to therapies in movement disorders is often measured by the use of clinician- and patient-reported outcomes. However, their use is limited by subjectivity, insensitivity to subtle changes, and recall bias [25]. Wearable digital devices often including accelerometers and gyroscopes are considered useful for the detection and monitoring of PD symptoms [26, 27] as they provide objective and accurate data and may be used in real-world settings [28, 29]. Ultimately, they should serve as reliable clinical decision support with regard to better targeting of interventions and improved patient selection for interventions [30]. Device-based assessment of gait and balance parameters showed high validity in comparison to more complex motion capture systems [31, 32] and can be applied with satisfactory feasibility in clinical settings [33]. Interestingly, gait parameters including gait speed may be altered up to four years before PD diagnosis [34] and gait analysis supported by machine learning can predict the risk of falling [35]. A recent cross-sectional study showed that such devices can also detect changes in PD symptoms due to treatment adaptation [36]. Regarding PD-MCT, a pilot study described improvements in various gait parameters including gait velocity and cadence in PD patients using a three-dimensional laboratory-based system of gait analysis [37]. However, overall evidence of changes in gait and balance parameters using wearable digital devices is scarce. We aimed at assessing these parameters as markers of response to PD-MCT.

In this exploratory observational study, we examined how digital device-based parameters of gait and balance correlate with clinical scores, how they changed throughout a two-week multidisciplinary inpatient PD treatment, and how changes in scores correlate with changes in device-based parameters.

## Methods

### Study design and participants

This exploratory analysis is part of an observational cohort study with a planned sample size of 94 PD patients (Park Move Study). The first 25 patients undergoing a two-week PD-MCT were included from September 2019 to April 2020 at the Department of Neurology at St. Josef-Hospital, Ruhr University Bochum, Germany. All patients gave written informed consent for inclusion before participation. The protocol was approved by the ethics committee of the Medical Faculty of the Ruhr University Bochum (Reg. Nr. 19-6659-MPG) and is listed in the German Clinical Trials Register (DRKS-ID: DRKS00020948). The Park Move Study will contribute harmonized data to the multicentre ComOn-Study [38] coordinated by UKSH University Hospital Kiel, Germany.

### Inclusion and exclusion criteria

We assessed all planned participants of inpatient PD-MCT for eligibility. Inclusion criteria were a minimum age of 18 years, capability for participation in device-based assessments, and a diagnosis of PD based on the UK Brain Bank Criteria and Movement Disorder Society (MDS) Clinical Diagnostic Criteria [39, 40]. Patients with secondary or atypical Parkinsonism were excluded as well as patients who refused participation or were not able to succeed in the gait analysis due to medical or mental conditions. Further exclusion criteria comprised a lack of consent, substance dependence (except nicotine) within 6 months before signing the consent form, history of stereotactic surgery, electroconvulsive therapy in the 180 days before screening, severe dementia based on a score of < 10 on the Mini-Mental State Examination (MMSE) test, acute psychotic disorder (benign hallucinations or previous psychotic episodes were no exclusion criteria), depression with suicidal ideation (previous episodes of major depression were no exclusion criteria), and illiteracy or insufficient language skills to complete the questionnaires.

### Procedures

**Fig. 1:**
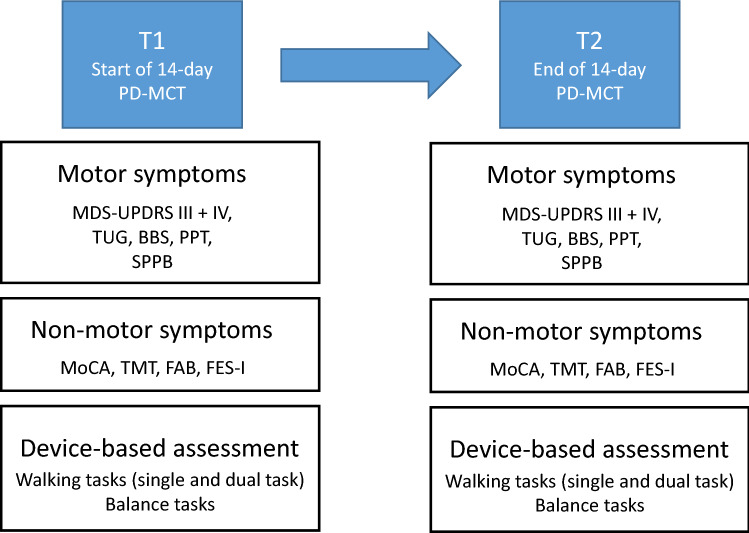
Study design and timings of assessment. *MDS-UPDRS* MDS-Unified Parkinson's Disease Rating Scale, *TUG* Timed up and Go Test, *BBS* Berg Balance Scale, *PPT* Purdue Pegboard Test, *FES-I* Falls efficacy scale, *SPPB* Short Physical Performance Battery, *MoCA* Montreal Cognitive Assessment, *TMT* Trail making test, *FAB* Frontal Assessment Battery

At the start (T1, baseline) and the end (T2) of the two-week PD-MCT, we assessed device-based parameters along with clinical scores, performed a clinical examination, and took the medical history of the patients. The first examination took place on day 1 or 2, the second examination on day 13 or 14 (Fig. 1).

### Gait and balance analysis

The patients were equipped with a wearable digital device system (RehaGait®, Hasomed, Magdeburg, Germany), placed at both ankles and the lower back (L5) by the investigators and connected with a tablet computer. They performed several supervised gait and balance tasks [41] during the ON medication state. Each task was usually performed once, but up to two repetitions (i.e., three runs) were possible if necessary due to disease severity, incorrect performance, or external interferences.

Walking tasks consisted of straight walking 20 m under single-task conditions at a normal, i.e., convenient, and fast pace, as well as under two dual-task conditions (motor-cognitive and motor-motor) with subtracting serial sevens from 659 (T1) or 829 (T2), and checking boxes, each time at a fast pace. During these tasks, the device recorded data from the built-in 3-axis accelerometer, 3-axis gyroscope, and 3-axis magnetometer which measured acceleration, angular velocity, and variations in the magnetic field, respectively. The raw data of the sensor placed at the lower back were afterwards processed through a validated algorithm [41] to obtain several parameters of gait and balance (for reviews see [42] and [43]) (Table 1).

**Table 1:** Device-based gait parameters and corresponding domains of gait [43, 44] analyzed in this study

Domain	Parameter	Description
Ambulatory activity	Gait velocity [m/s]	Calculated by dividing 20 m by the ambulation time
	Step count	Number of steps needed to walk 20 m
	Cadence [steps/s]	Number of steps per second
Pace	Stride length [cm]	Distance between two heel strikes of the same foot; one stride corresponds to one gait cycle or to two steps
Rhythm	Stride time [s]	Time elapsed between two heel strikes of the same foot
	Step time [s]	Time elapsed between the heel strikes of one and the opposite foot
	Stance time [s]	Time required for the stance phase, i.e., between heel strike and toe-off of the same foot
	Swing time [s]	Time required for the swing phase, i.e., between toe-off and heel strike of the same foot
	Double limb support time [s]	Time required for the periods where both feet are on the ground during one gait cycle

**Fig. 2:**
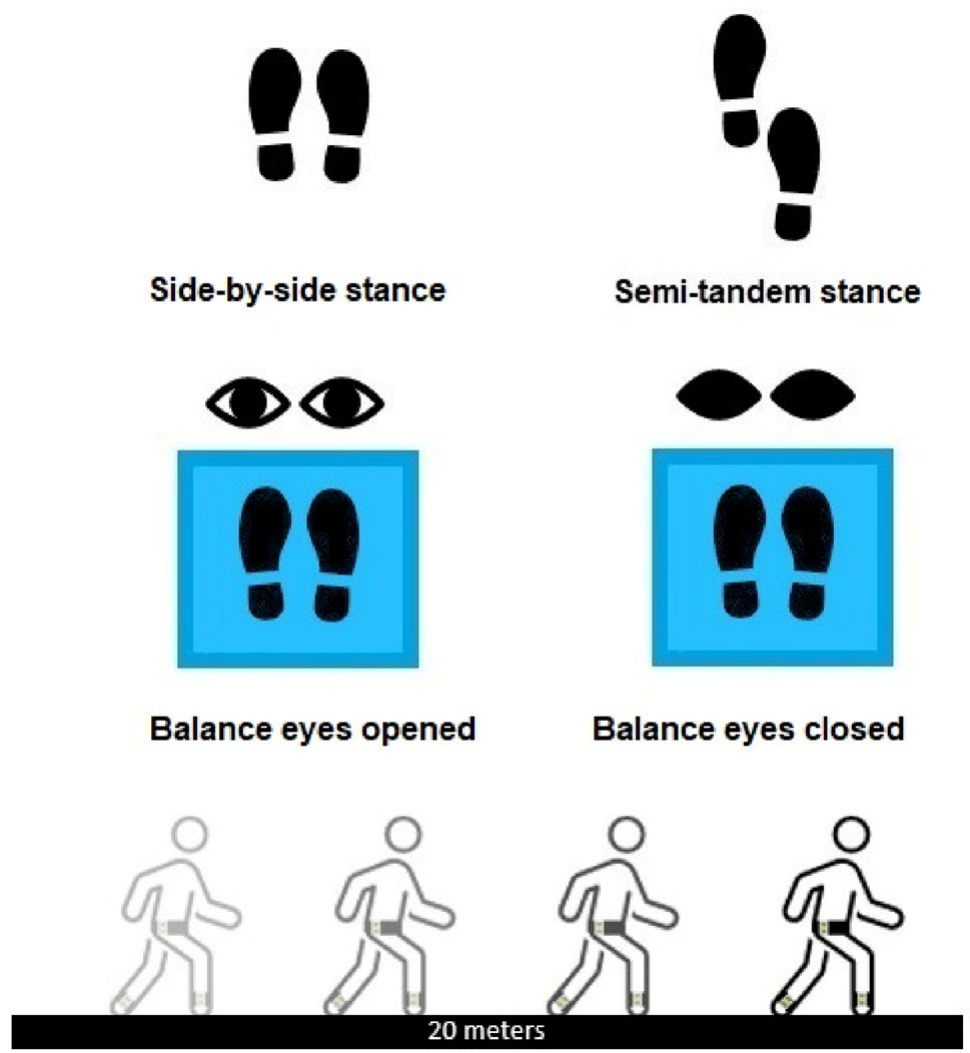
Balance tasks and settings of walking tasks; Side-by-side stance, semi-tandem stance, balance task with opened and closed eyes, respectively; 20-m track for straight walking under single-task (normal and fast pace) and two dual-task conditions (checking boxes, subtracting serial sevens)

Balance tasks consisted of a side-by-side and semi-tandem stance for 10 s each, and standing on a balance mat (Airex® Balance Pad, 48 × 40 × 6 cm) with opened and closed eyes for 30 s each [45], as displayed in Fig. 2. During these tasks, the sway area was determined (unit: mm^2^/s^4^). This is the 95% confidence ellipse of planar acceleration at the L5 level along the anterior–posterior and medio-lateral axis, enclosed by the trajectory of the center of pressure.

### Clinical Scores and Questionnaires

Motor symptoms and mobility, as well as physical capabilities, were recorded using the MDS Unified Parkinson’s Disease Rating Scale (MDS-UPDRS) parts III and IV [46], Berg Balance Scale (BBS) [47], Timed Up and Go Test (TUG) [48], Purdue Pegboard Test (PPT) [49–51] and Short Physical Performance Battery (SPPB) [52, 53]. Disease severity was assessed by the modified Hoehn and Yahr scale [53, 54].

For assessing non-motor symptoms, we used the Montreal Cognitive Assessment (MoCA) [55], Trail Making Test (TMT) [56], and the Frontal Assessment Battery (FAB) [57, 58]. Further, we applied the Falls Efficacy Scale (FES-I) to determine the fear of falling [59, 60].

### Intervention

PD-MCT is a multidisciplinary inpatient short-term rehabilitation, which has been described in detail previously [18, 19]. It globally aims at optimizing functional capacity and reducing disability, thereby promoting quality of life. It is co-defined by specific formal requirements of the German reimbursement system. PD-MCT is directed by the principles of individualized [23] and person-centered [24] health care. Thus, it centrally considers clinical subtypes, personality, lifestyle, or comorbidities in the provision of care and is guided by the individual's values and preferences, i.e., the individual therapy goals.

Along with adjustments to pharmacotherapy, several non-pharmacologic therapies such as physiotherapy, occupational therapy, speech and language therapy, and specialized nurse care are conducted (at least 7.5 h per week). Examples of therapies applied include amplitude-oriented therapies (e.g., Lee Silverman Voice Treatment (LSVT) BIG [61] and LSVT LOUD [62]), everyday life-oriented therapies (e.g., training of turning in bed or raising from a chair), the training of focused attention in everyday activities, and the application of cognitive strategies in performing the execution of movements. Therapies orient themselves on the available PD guidelines for physiotherapy [63], speech and language therapy [64], and occupational therapy [65].

In our department, the duration of the inpatient stay is 14 days. Before the hospital stay, the suitability for PD-MCT and individual therapy goals are usually assessed during an outpatient visit. In some cases, crisis situations such as exacerbations of motor and non-motor symptoms can lead to admission with a secondary application of PD-MCT. Upon admission, each therapeutic discipline assesses the overall condition of the person living with PD and identifies core problems. Individual therapy goals are defined in consultation with the patient together with caregivers and are discussed and adjusted during the therapy. The baseline assessment is followed by targeted interventions that are tailored to the individual’s needs in terms of content, frequency, and intensity. In more detail, usually 4.5, 3.5, 2.5, and ca. 4 h of physiotherapy, occupational therapy, speech and language therapy, and exercise, respectively, are applied throughout the two-week PD-MCT in our department [19], making a total of 14.5 h. At the end of the therapy, a final discussion is held with the patients and their caregivers to achieve the longest possible lasting effect of the therapy carried out in everyday life.

### Statistical analysis

Results were analyzed with IBM SPSS Version 27. Normal distribution was tested with the Shapiro–Wilk test and Q–Q-Plots. Parameters “Step count” and “Stride length” were normalized for a velocity of 1 m/s for all participants as these parameters have repeatedly been shown to correlate significantly with gait velocity [66]. For the checking boxes walking task, both original and corrected step count and stride length were included in the analyses. Outcomes were visualized by boxplots and scatterplots. Correlations were analyzed exploratively using Spearman’s rank correlation coefficient. For comparison of the parameters at T1 and T2, Bayes factors (BF_10_) [67] and *P* values were calculated using a Bayesian *t* test. *P* values below 0.05 were considered significant. We reported only raw *P* values. For comparisons (Tables 4 and S2), Bonferroni correction for multiple testing was applied by dividing the alpha level by the number of tests conducted regarding the same hypothesis, or walking paradigm and balance parameter, respectively. The factors of correction were indicated below the respective tables and the *P* values considered significant after Bonferroni-correction were additionally marked by typographical notes.

## Results

During the period between September 2019 and April 2020, 43 patients participating in PD-MCT were assessed for eligibility, and 25 patients met the inclusion criteria. All included patients were assessed at T1 and completed the assessment at T2. Exclusions due to not-fulfilled selection criteria occurred mostly because of a diagnosis of atypical Parkinsonism.

**Fig. 3:**
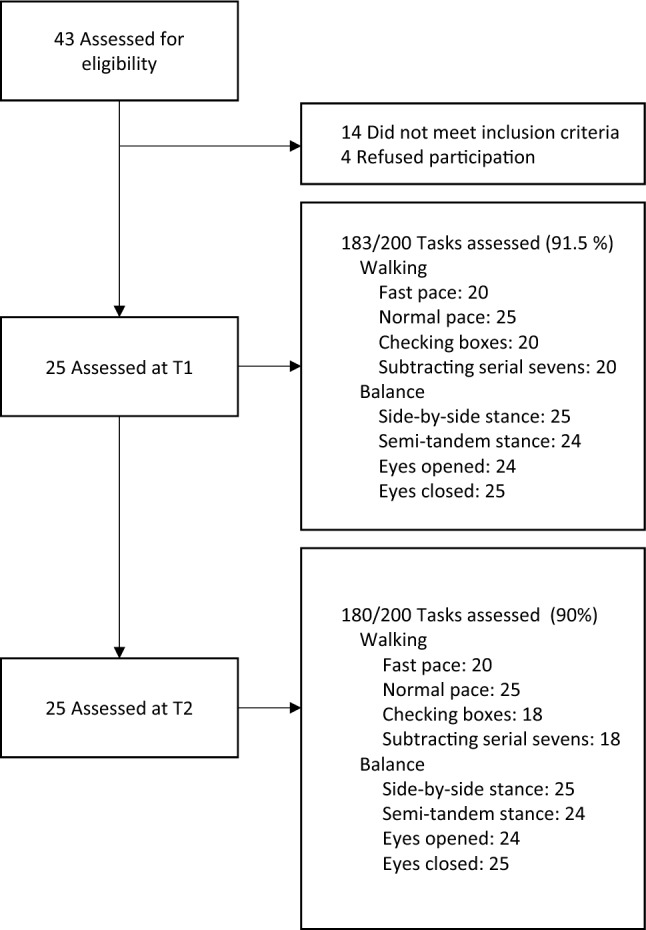
Study flow chart

Missing data can be derived from Tables 3 and 4 and Fig. 3. Owing to disease severity, five subjects could not perform the dual-tasking and the fast pace walking tasks at neither T1 nor T2. Additionally, due to technical reasons (loss of Bluetooth connection between the tablet computer and the wearable device), four datasets of dual-task walking tasks concerning three subjects at T2 were not recorded (Table 4, Fig. 3).

The study population characteristics are displayed in Table 2.

**Table 2:** Study population characteristics

Variable	*M*	SD
Age, *a*	66.9	9.9
Sex, female/male, *n* (%)	7/18	(28/72)
Duration of disease, *a*	8.64	5.25
Hoehn and Yahr, median (IQR)	2.5	2–3
1	2	(8)
2	5	(20)
2.5	8	(32)
3	9	(36)
4	1	(4)
MDS-UPDRS III (0–132)	32.5	14.9
MDS-UPDRS IV (0–24)	6.3	4.7
TUG, s	10.9	4.7
PPT R + L + B	27.5	6.9
BBS (0–56)	46.9	8.0
FES-I (16–64)	26.9	10.1
MoCA (0–30)	24.7	2.9
FAB (0–18)	15.4	1.9
ΔTMT, s	74.8	55.7
SPPB (0–12)	8.2	2.3
LED, mg	651	428

*MDS-UPDRS III* Movement Disorder Society Unified Parkinson’s Disease Rating Scale Part III: motor examination, *MDS-UPDRS IV* Part IV: motor complications, *TUG* Timed-up-and-go test*, PPT* Purdue Pegboard Test, *R* total number of sticks inserted with right, *L* left, *B* both hands, *BBS* Berg Balance Scale, *MoCA* Montreal Cognitive Assessment, *FAB* Frontal Assessment Battery, *TMT* Trail Making Test, *FES-I* Falls Efficacy Scale International, *SPPB* Short Physical Performance Battery*, LED* Daily Levodopa equivalent dose

### Correlations of device-based parameters with clinical scores at baseline

Both a slow gait and a large sway area were associated with higher motor severity on MDS-UPDRS III, more fear of falling on FES-I, less functional capacity of the lower extremities scored by SPPB, lower balance scores on BBS, and with lower functional mobility as measured by the TUG test (Table 3). This applies to all walking tasks regarding gait velocity and to specific balance tasks regarding sway area (Table 3). The balance tasks showing significant associations of sway area with clinical scores always included semi-tandem stance, and additionally the eyes-opened balance task for MDS-UPDRS III, and eyes-closed balance task/side-by-side stance for both FES-I and SPPB (Table 3).

**Table 3:** Correlations of device-based parameters with clinical scores

Spearman's *r*_s_	MDS-UPDRS III	MDS-UPDRS IV	PPT	TUG	BBS	FES-I	MoCA	FAB	TMT	SPPB
Clinical scores, *n* = 25										
MDS-UPDRS III	–									
MDS-UPDRS IV	0.49*	–								
PPT	− 0.48*	− 0.09	–							
TUG	0.69**	0.47*	− 0.44*	–						
BBS	− 0.65**	− 0.38	0.49*	− 0.76**	–					
FES-I	0.51**	0.41*	− 0.44*	0.72**	− 0.55**	–				
MoCA	− 0.39	− 0.27	0.37	− 0.45*	0.48*	− 0.42*	–			
FAB	− 0.52**	− 0.44*	0.41*	− 0.51**	0.42*	− 0.51**	0.7**	–		
TMT	0.40	0.16	− 0.30	0.34	− 0.41*	0.24	− 0.71**	− 0.54**	–	
SPPB	− 0.63**	− 0.42*	0.51**	− 0.72**	0.85**	− 0.55**	0.30	0.36	− 0.15	–
Straight walk fast pace, *n* = 20										
Velocity [m/s]	− 0.50*	− 0.31	0.44	− 0.81**	0.68**	− 0.75**	0.46*	0.44*	− 0.37	0.74**
Step count	− 0.08	0.54*	0.16	− 0.19	0.19	− 0.18	0.23	0.17	− 0.37	− 0.05
Cadence [Steps/s]	− 0.11	0.47*	0.11	− 0.13	0.09	− 0.14	0.16	0.12	− 0.22	− 0.11
Stride length [cm]	0.12	− 0.38	− 0.03	0.08	− 0.04	0.00	− 0.15	− 0.10	0.17	0.15
Stride time [s]	0.19	− 0.49*	− 0.22	0.28	− 0.27	0.26	− 0.25	− 0.16	0.32	− 0.07
Step time [s]	0.19	− 0.50*	− 0.20	0.27	− 0.26	0.26	− 0.25	− 0.14	0.31	− 0.06
Stance time [s]	0.22	− 0.43	− 0.23	0.32	− 0.31	0.27	− 0.27	− 0.21	0.35	− 0.08
Swing time [s]	0.25	− 0.46*	− 0.12	0.22	− 0.20	0.11	− 0.18	− 0.02	0.26	− 0.05
Double limb support time [s]	0.19	− 0.41	− 0.20	0.30	− 0.31	0.27	− 0.28	− 0.20	0.34	− 0.03
Straight walk normal pace, *n* = 25										
Velocity [m/s]	− 0.60**	− 0.46*	0.42*	− 0.80**	0.70**	− 0.66**	0.24	0.31	− 0.07	0.86**
Step count	− 0.01	0.46*	0.17	− 0.06	0.06	0.27	− 0.09	0.01	0.09	− 0.02
Cadence [steps/s]	− 0.16	0.39	0.13	− 0.13	0.13	0.23	− 0.13	− 0.06	0.12	0.08
Stride length [cm]	0.24	− 0.34	− 0.09	0.16	− 0.19	− 0.18	0.10	0.09	− 0.11	− 0.14
Stride time [s]	0.21	− 0.32	− 0.08	0.22	− 0.21	− 0.18	0.08	0.04	− 0.06	− 0.15
Step time [s]	0.21	− 0.32	− 0.08	0.23	− 0.21	− 0.17	0.10	0.04	− 0.07	− 0.16
Stance time [s]	0.27	− 0.28	− 0.13	0.27	− 0.26	− 0.13	0.07	0.00	− 0.07	− 0.20
Swing time [s]	0.20	− 0.37	− 0.09	0.18	− 0.19	− 0.20	0.03	0.02	0.03	− 0.14
Double limb support time [s]	0.24	− 0.24	− 0.08	0.28	− 0.26	− 0.15	0.08	− 0.01	− 0.06	− 0.19
Straight walk checking boxes, *n* = 20										
Velocity [m/s]	− 0.53*	− 0.34	0.72**	− 0.64**	0.57**	− 0.48*	0.36	0.32	− 0.24	0.80**
Step count	− 0.04	0.49*	0.23	− 0.12	0.13	− 0.13	0.06	− 0.01	− 0.11	0.13
Cadence [steps/s]	− 0.05	0.48*	0.31	− 0.11	0.14	− 0.11	− 0.03	− 0.13	− 0.09	0.13
Stride length [cm]	− 0.07	− 0.50*	− 0.25	0.03	− 0.03	0.03	0.07	0.13	0.06	− 0.03
Stride time [s]	0.09	− 0.44	− 0.35	0.22	− 0.22	0.16	− 0.07	0.04	0.17	− 0.22
Step time [s]	0.11	− 0.41	− 0.32	0.24	− 0.22	0.15	− 0.09	0.04	0.18	− 0.22
Stance time [s]	0.08	− 0.43	− 0.36	0.18	− 0.23	0.15	− 0.05	0.09	0.17	− 0.20
Swing time [s]	0.21	− 0.38	− 0.32	0.33	− 0.35	0.16	− 0.25	− 0.08	0.27	− 0.29
Double limb support time [s]	0.04	− 0.45*	− 0.33	0.13	− 0.17	0.12	0.01	0.15	0.11	− 0.15
Straight walk subtracting serial sevens, *n* = 20										
Velocity [m/s]	− 0.52*	− 0.19	0.77**	− 0.65**	0.64**	− 0.50*	0.42	0.33	− 0.33	0.85**
Step count	− 0.01	0.31	0.11	− 0.29	0.09	0.07	0.31	0.38	− 0.26	0.01
Cadence [steps/s]	− 0.09	0.26	0.12	− 0.28	0.08	0.11	0.25	0.28	− 0.21	0.04
Stride length [cm]	0.13	− 0.27	− 0.07	0.23	− 0.05	− 0.11	− 0.23	− 0.26	0.19	0.01
Stride time [s]	0.11	− 0.29	− 0.15	0.28	− 0.10	− 0.11	− 0.25	− 0.29	0.23	− 0.05
Step time [s]	0.11	− 0.29	− 0.16	0.29	− 0.12	− 0.11	− 0.27	− 0.31	0.25	− 0.05
Stance time [s]	0.09	− 0.24	− 0.15	0.30	− 0.12	− 0.08	− 0.28	− 0.33	0.25	− 0.06
Swing time [s]	0.15	− 0.32	− 0.06	0.25	− 0.02	− 0.11	− 0.21	− 0.25	0.23	0.02
Double limb support time [s]	0.02	− 0.15	− 0.12	0.30	− 0.13	− 0.11	− 0.27	− 0.34	0.27	− 0.06
Sway area [mm^2^/s^4^], *n* = 25										
Side-by-side stance	0.27	0.43*	− 0.26	0.35	− 0.25	0.43*	0.08	− 0.24	− 0.36	− 0.46*
Semi-tandem stance, *n* = 24	0.52**	0.28	− 0.45*	0.48*	− 0.63**	0.61**	− 0.40	− 0.46*	0.25	− 0.50*
Balance eyes opened, *n* = 24	0.55**	0.39	− 0.53**	0.32	− 0.35	0.32	− 0.34	− 0.39	0.00	− 0.32
Balance eyes closed	0.21	0.42*	− 0.19	0.39	− 0.36	0.42*	− 0.20	− 0.30	0.11	− 0.41*

*p < 0.05

**p < 0.01

**Fig. 4:**
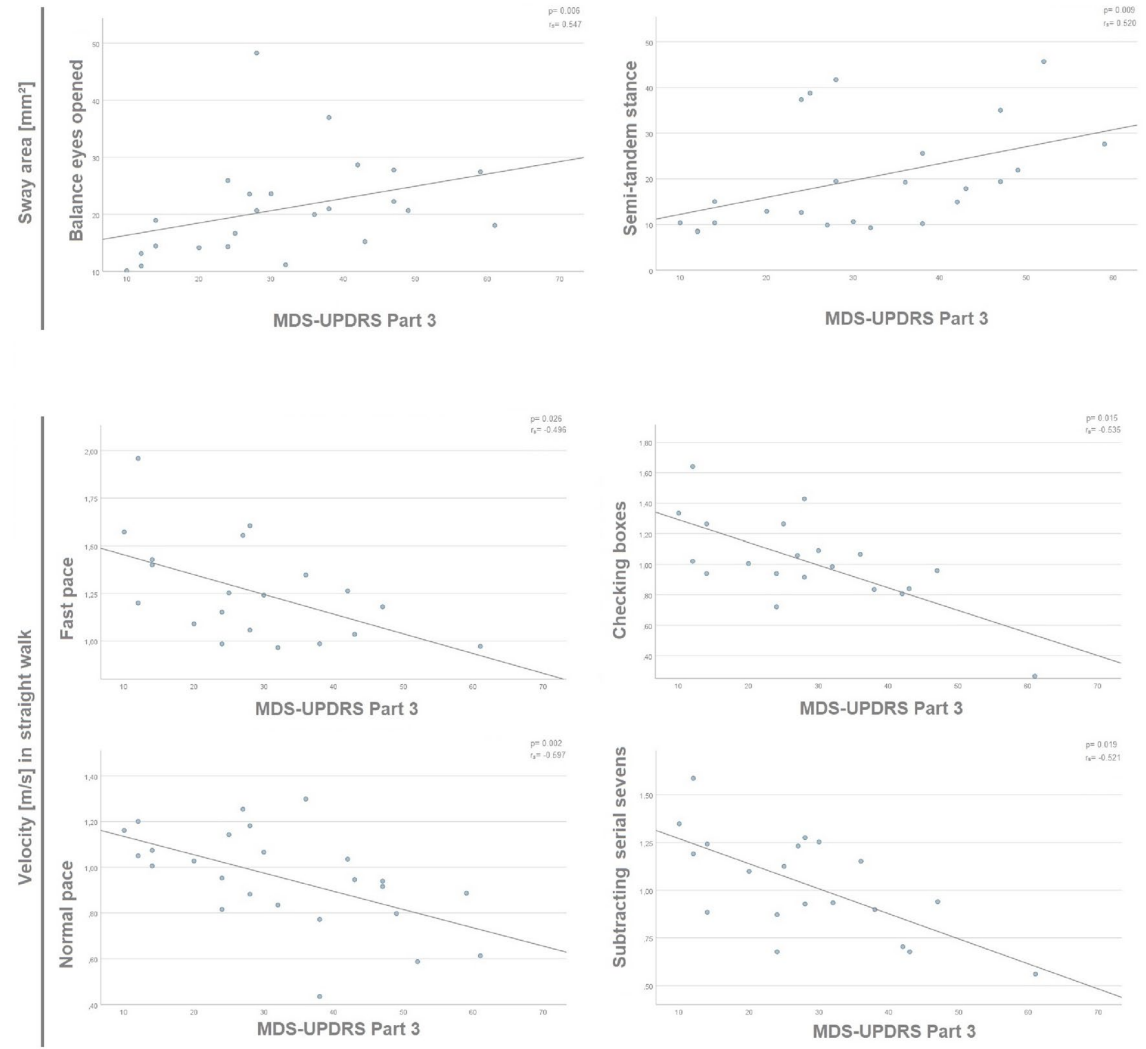
Correlations of MDS-UPDRS III with device-based parameters

From the clinical perspective, MDS-UPDRS III negatively correlated with gait velocity in all walking tasks and showed a positive association with sway area in semi-tandem stance and eyes-opened balance task (Fig. 4, Table 3).

The MDS-UPDRS IV score correlated significantly with several device-based parameters. It showed a positive association with step count in walking at a fast and normal pace as well as in the checking boxes task (Table 3). Accordingly, there were negative correlations between MDS-UPDRS IV and stride length in the same walking tasks, albeit statistically significant in the checking boxes task only (Table 3). However, MDS-UPDRS IV significantly negatively correlated with stride length in these tasks if the parameter was not corrected for velocity (Table S1). Like MDS-UPDRS III, MDS-UPDRS IV correlated with the sway area in two of the balance tasks, specifically in the side-by-side stance and eyes-closed task (Table 3).

### Differences between T1 and T2

Throughout PD-MCT, gait velocity increased in all walking tasks (Table 4). In addition, step count decreased significantly in the walking while checking boxes paradigm. When not corrected for velocity, stride length analogously increased significantly during motor-motor dual-tasking (Table S2). However, after accounting for multiple testing, the only device-based parameter changed was gait velocity under dual-task conditions. Other device-based parameters such as double-support time showed a trend towards significant change (Table 4).

**Table 4:** Changes in device-based parameters of gait and balance and clinical scores after Parkinson’s Disease Multimodal Complex Treatment

Variable	T1		T2		Δ T2–T1			
	*M*	SD	*M*	SD	*M*	SE	BF_10_	*p*
MDS-UPDRS III	32.5	14.9	26.6	11.9	− 5.9	1.3	221.70	< 0.001*
MDS-UPDRS IV	6.3	4.7	4.1	2.8	− 2.2	0.9	2.65	**0.016**
TUG [s]	10.9	4.7	9.8	3.5	− 1.1	0.5	1.70	**0.028**
PPT R + L + B	27.5	6.9	30.7	6.5	3.2	0.7	156.19	< 0.001*
BBS	46.9	8.0	49.4	7.4	2.4	0.5	188.37	< 0.001*
MoCA	24.7	2.9	24.6	2.4	− 0.1	0.5	0.16	0.873
FAB	15.4	1.9	16.6	1.7	1.2	0.3	53.55	0.001*
ΔTMT [s]	74.8	55.7	76.1	58.5	1.3	11.0	0.15	0.906
FES-I	26.9	10.1	26.7	10.2	− 0.2	1.2	0.16	0.866
SPPB	8.2	2.3	8.6	2.2	0.4	0.3	0.50	0.123
LED [mg]	656	399	833	451	177	55	10.88	0.003*
Straight walk fast pace, *n* = 20								
Velocity [m/s]	1.26	0.26	1.34	0.32	0.07	− 0.13	2.84	**0.017**
Step count	26.1	8.6	23.0	7.5	− 3.1	− 9.6	0.44	0.168
Cadence [steps/s]	1.3	0.5	1.4	0.5	0.0	− 0.6	0.18	0.716
Stride length [cm]	88.4	42.7	100.3	39.3	11.9	− 47.6	0.30	0.280
Stride time [s]	1.44	0.48	1.37	0.47	− 0.07	− 0.51	0.20	0.547
Step time [s]	0.72	0.24	0.68	0.24	− 0.04	− 0.26	0.21	0.517
Stance time [s]	1.25	0.39	1.19	0.39	− 0.05	− 0.43	0.20	0.594
Swing time [s]	0.20	0.10	0.18	0.09	− 0.02	− 0.09	0.29	0.294
Double limb support time [s]	0.52	0.15	0.51	0.15	− 0.02	− 0.17	0.19	0.675
Straight walk normal pace, *n* = 25								
Velocity [m/s]	0.96	0.21	1.01	0.20	0.05	− 0.10	2.51	**0.018**
Step count	26.6	9.2	29.3	8.7	2.7	− 12.4	0.27	0.289
Cadence [steps/s]	1.3	0.4	1.4	0.4	0.1	− 0.5	0.25	0.330
Stride length [cm]	89.2	32.6	82.6	33.0	− 6.6	− 38.6	0.22	0.401
Stride time [s]	1.56	0.58	1.41	0.48	− 0.15	− 0.71	0.26	0.306
Step time [s]	0.78	0.29	0.71	0.24	− 0.07	− 0.35	0.25	0.316
Stance time [s]	1.36	0.49	1.23	0.39	− 0.13	− 0.60	0.26	0.295
Swing time [s]	0.20	0.10	0.18	0.09	− 0.02	− 0.11	0.24	0.350
Double limb support time [s]	0.57	0.20	0.52	0.15	− 0.05	− 0.25	0.25	0.322
Straight walk checking boxes, *n* = 18								
Velocity [m/s]	1.04	0.30	1.11	0.28	0.07	− 0.09	12.65	**0.003**†
Step count	32.3	15.6	22.7	8.1	− 9.6	− 16.4	2.30	**0.023**
Cadence [steps/s]	1.4	0.5	1.1	0.5	− 0.3	− 0.6	0.91	0.070
Stride length [cm]	82.8	37.2	101.2	37.0	18.4	− 43.3	0.75	0.090
Stride time [s]	1.45	0.53	1.67	0.52	0.23	− 0.54	0.73	0.092
Step time [s]	0.72	0.27	0.84	0.26	0.11	− 0.27	0.75	0.090
Stance time [s]	1.25	0.43	1.45	0.43	0.20	− 0.47	0.73	0.093
Swing time [s]	0.19	0.10	0.23	0.10	0.04	− 0.08	0.84	0.077
Double limb support time [s]	0.53	0.17	0.61	0.17	0.08	− 0.19	0.67	0.103
Straight walk subtracting serial sevens, *n* = 18								
Velocity [m/s]	1.03	0.28	1.10	0.26	0.07	− 0.09	16.52	**0.002**†
Step count	26.8	9.3	26.5	8.9	− 0.4	− 8.9	0.18	0.859
Cadence [steps/s]	1.2	0.4	1.3	0.5	0.0	− 0.5	0.19	0.773
Stride length [cm]	93.3	35.8	92.1	38.2	− 1.2	− 35.3	0.18	0.889
Stride time [s]	1.58	0.46	1.54	0.48	− 0.04	− 0.47	0.19	0.710
Step time [s]	0.79	0.23	0.77	0.24	− 0.02	− 0.23	0.19	0.689
Stance time [s]	1.37	0.37	1.34	0.39	− 0.03	− 0.39	0.19	0.712
Swing time [s]	0.22	0.10	0.21	0.10	− 0.01	− 0.09	0.20	0.648
Double limb support time [s]	0.58	0.14	0.56	0.15	− 0.01	− 0.15	0.19	0.722
Sway area [mm^2^/s^4^], *n* = 25								
Side-by-side stance	14.4	5.4	15.6	7.2	1.2	− 5.54	0.28	0.271
Semi-tandem stance, *n* = 24	20.1	11.6	19.6	17.5	− 0.5	− 19.39	0.16	0.892
Balance eyes opened, *n* = 24	21.0	8.8	21.4	6.8	0.4	− 10.09	0.16	0.856
Balance eyes closed	98.8	93.3	68.8	36.3	− 30.0	− 87.12	0.60	0.098

Significant changes are highlighted in bold

*BF*_*10*_ Bayesian factor

*Significant at Bonferroni-adjusted alpha level of 0.005 (factor of correction (CF): 11)

^†^Significant at adjusted alpha level of 0.006 (CF: 9)

Regarding clinical scores, highly significant improvements occurred in motor severity (MDS-UPDRS III), balance (BBS), dexterity (PPT), and executive functions (FAB; Table 4). Additionally, significant changes in motor complications (MDS-UPDRS IV) and functional mobility (TUG) were recorded. Throughout the treatment, there was a significant increase in the daily Levodopa equivalent dose (Table 4). At T2, scores on FES-I, TMT, MoCA, and the SPPB were not altered significantly from T1.

### Significant correlation of changes

Analyses revealed significant positive correlations of changes in MDS-UPDRS IV with changes in step count and cadence in walking at a normal and fast pace, respectively (Table 5). In parallel, changes in MDS-UPDRS IV significantly negatively correlated with changes in stride length during walking at a normal pace if the latter was not corrected for velocity (Table S3). A decrease in MDS-UPDRS III was associated with an increase in the sway area during side-by-side stance (Table 5).

**Table 5:** Correlations of changes in device-based parameters with those in clinical scores

Spearman’s *r*_s_	Δ MDS-UPDRS III	Δ MDS-UPDRS IV	Δ PPT	Δ TUG	Δ BBS	Δ FES-I	Δ MoCA	Δ FAB	Δ TMT	Δ SPPB	Δ LED
Δ MDS-UPDRS III	–										
Δ MDS-UPDRS IV	− 0.04	–									
Δ PPT	0.20	0.06	–								
Δ TUG	0.39	0.07	0.18	–							
Δ BBS	− 0.48*	0.18	0.17	− 0.50*	–						
Δ FES-I	0.04	− 0.15	0.02	0.29	0.08	–					
Δ MoCA	− 0.12	0.03	− 0.17	− 0.38	0.36	− 0.02	–				
Δ FAB	− 0.17	− 0.30	0.04	− 0.25	0.04	0.20	0.09	–			
Δ TMT	0.41*	− 0.14	− 0.16	0.37	− 0.47*	0.03	− 0.08	0.17	–		
Δ SPPB	− 0.20	0.22	− 0.03	− 0.02	0.22	− 0.11	− 0.02	− 0.08	− 0.20	–	
Δ LED	0.04	0.16	0.14	− 0.35	0.23	− 0.21	0.27	− 0.14	− 0.29	− 0.18	–
Straight walk fast pace, *n* = 20											
Δ Velocity	0.06	− 0.39	− 0.12	− 0.40	− 0.09	− 0.09	0.07	− 0.17	− 0.20	− 0.42	0.37
Δ Step count	− 0.14	0.30	0.00	0.29	− 0.15	− 0.21	− 0.36	− 0.01	− 0.07	0.13	− 0.22
Δ Cadence	− 0.17	0.47*	0.01	0.23	− 0.32	− 0.24	− 0.30	− 0.26	− 0.11	− 0.04	0.05
Δ Stride length	0.06	− 0.08	0.16	− 0.21	0.15	0.09	0.20	0.05	0.02	− 0.01	0.24
Δ Stride time	0.18	− 0.37	0.14	− 0.14	0.27	0.24	0.17	0.24	− 0.05	0.14	0.04
Δ Step time	0.14	− 0.37	0.18	− 0.15	0.26	0.20	0.14	0.25	− 0.09	0.13	0.07
Δ Stance time	0.16	− 0.39	0.13	− 0.09	0.26	0.31	0.16	0.27	0.00	0.17	0.00
Δ Swing time	0.20	− 0.37	0.14	− 0.16	0.20	0.06	0.01	0.17	− 0.07	− 0.12	0.15
Δ Double limb support time	0.12	− 0.35	0.16	− 0.11	0.30	0.31	0.22	0.27	− 0.02	0.19	0.05
Straight walk normal pace, *n* = 25											
Δ Velocity	− 0.08	− 0.13	− 0.22	− 0.39	− 0.09	− 0.15	0.06	0.16	− 0.08	− 0.01	0.21
Δ Step count	0.14	0.48*	− 0.11	0.10	− 0.24	0.07	− 0.07	− 0.17	0.16	− 0.14	0.27
Δ Cadence	0.12	0.26	− 0.10	− 0.07	− 0.19	0.12	0.04	− 0.01	0.20	− 0.12	0.29
Δ Stride length	− 0.03	− 0.28	0.09	0.05	0.17	− 0.22	0.00	− 0.02	− 0.19	0.22	− 0.26
Δ Stride time	− 0.09	− 0.15	0.16	0.13	0.18	− 0.14	− 0.05	− 0.08	− 0.26	0.15	− 0.28
Δ Step time	− 0.09	− 0.17	0.17	0.12	0.18	− 0.12	− 0.06	− 0.06	− 0.28	0.12	− 0.30
Δ Stance time	− 0.07	− 0.16	0.22	0.14	0.18	− 0.08	− 0.05	0.01	− 0.18	0.13	− 0.33
Δ Swing time	0.02	− 0.29	0.10	0.05	0.12	− 0.23	− 0.09	− 0.09	− 0.32	0.19	− 0.22
Δ Double limb support time	− 0.08	− 0.10	0.25	0.11	0.19	− 0.06	− 0.01	0.04	− 0.14	0.13	− 0.37
Straight walk checking boxes, *n* = 18											
Δ Velocity	− 0.06	− 0.21	0.39	− 0.38	0.28	0.35	0.33	0.42	− 0.30	− 0.01	0.13
Δ Step count	− 0.05	0.07	− 0.41	0.09	− 0.06	− 0.56*	0.24	− 0.44	0.29	0.00	0.33
Δ Cadence	0.19	0.35	0.02	0.19	− 0.23	− 0.53*	0.04	− 0.31	0.45	− 0.17	0.32
Δ Stride length	− 0.19	− 0.33	− 0.03	− 0.14	0.09	0.45	0.08	0.27	− 0.31	0.24	− 0.30
Δ Stride time	− 0.15	− 0.46	− 0.16	− 0.20	0.16	0.49*	− 0.16	0.44	− 0.30	0.22	− 0.29
Δ Step time	− 0.16	− 0.47	− 0.18	− 0.22	0.16	0.47*	− 0.14	0.44	− 0.27	0.22	− 0.30
Δ Stance time	− 0.23	− 0.36	− 0.05	− 0.21	0.28	0.46	− 0.08	0.44	− 0.39	0.35	− 0.33
Δ Swing time	− 0.05	− 0.31	− 0.20	− 0.06	− 0.06	0.46	− 0.24	0.46	− 0.20	0.19	− 0.43
Δ Double limb support time	− 0.18	− 0.39	− 0.01	− 0.24	0.33	0.53*	0.00	0.38	− 0.37	0.39	− 0.22
Straight walk subtracting seven, *n* = 18											
Δ Velocity	− 0.37	− 0.08	0.11	− 0.33	0.27	0.26	− 0.02	− 0.03	− 0.32	− 0.23	0.24
Δ Step count	− 0.21	0.24	− 0.01	0.03	− 0.09	0.24	0.32	0.19	0.05	− 0.04	0.16
Δ Cadence	− 0.14	0.07	0.19	0.02	− 0.06	0.51*	0.13	0.19	− 0.14	− 0.18	0.36
Δ Stride length	0.31	− 0.05	0.05	− 0.04	0.01	− 0.46	0.02	− 0.06	0.07	0.18	− 0.36
Δ Stride time	0.35	− 0.09	− 0.17	0.00	− 0.12	− 0.56*	− 0.18	− 0.15	0.19	0.06	− 0.23
Δ Step time	0.35	− 0.09	− 0.17	0.00	− 0.12	− 0.56*	− 0.18	− 0.15	0.19	0.06	− 0.23
Δ Stance time	0.25	− 0.12	− 0.15	− 0.02	− 0.17	− 0.62**	− 0.30	− 0.13	0.20	0.08	− 0.26
Δ Swing time	0.41	− 0.15	− 0.13	− 0.07	0.01	− 0.39	− 0.11	− 0.14	0.11	0.04	− 0.09
Δ Double limb support time	0.22	− 0.13	− 0.13	− 0.05	− 0.16	− 0.64**	− 0.37	− 0.19	0.17	0.04	− 0.22
Sway area											
Δ Side-by-side stance, *n* = 25	− 0.55**	− 0.10	− 0.13	− 0.29	0.17	0.06	0.21	0.20	− 0.15	− 0.25	− 0.33
Δ Semi-tandem stance, *n* = 24	0.04	− 0.10	0.09	0.06	− 0.06	− 0.02	0.06	0.09	0.00	− 0.10	0.22
Δ Balance eyes opened, *n* = 24	− 0.03	− 0.02	0.06	0.29	− 0.09	0.00	0.14	− 0.18	− 0.08	− 0.16	− 0.11
Δ Balance eyes closed, *n* = 25	− 0.15	0.25	0.00	0.23	0.08	0.30	0.28	0.01	0.01	− 0.34	0.18

*p < 0.05

**p < 0.01

Several significant correlations were found between the change in fear of falling on FES-I and changes in device-based parameters for dual-task walking paradigms (Table 5).

In the subtracting serial sevens walking task, a decrease in fear of falling was associated with fewer steps per second (cadence), and longer times required for one stride, one step, the stance, and the double support phase (Table 5). Of note, the strongest associations were found for longer stance and double support phases.

Conversely, in the checking boxes walking task, a decrease in fear of falling throughout PD-MCT was associated with more steps per second, and shorter times required for one stride, one step, and the double-support phase (Table 5).

## Discussion

To determine correlations between device-based parameters of gait and balance and clinical scores, their changes throughout the inpatient multidisciplinary PD-MCT, and correlations between their changes, an exploratory observational study was conducted.

### Baseline correlations

Both reduced gait velocity and larger sway area were associated with higher disease severity. As expected, the mean values of gait velocity in this study were smaller than in healthy males [68] and correspond to normative data in early-stage PD during convenient walking [69]. We were thus able to confirm earlier findings from cross-sectional studies that showed a negative association between gait speed and (motor) disease stage in PD [69–73]. Analogously, larger sway areas at more severe disease stages have been described previously [74, 75].

Interestingly, patients with more severe motor complications (motor fluctuations or dyskinesia) took shorter steps during the dual-task motor-walk paradigm than patients with milder motor complications. This association may be of precedential interest for future clinical decision-making (see below). It has already been shown that PD per se [76], advanced disease stage [69, 70], PIGD phenotype [77], medication OFF states [78–80], and dual-tasking with cognitive load [81–84] are associated with shortened step and stride lengths. Dual-tasking abilities are particularly relevant to daily life and may predict daily functioning in PD [85]. Cognitive loading accelerates gait deficits probably due to concurrent recruitment of neural capacities [83]. This neural overload is making dual-task gait more challenging for affected individuals than single-task gait and is possibly rendering motor-motor dual-tasking more sensitive to PD gait deficits than motor-cognitive dual-tasking [86]. This could explain the detection of associations between motor complications and shortened steps during motor-motor dual-task gait assessment, although the small sample size limits the generalizability.

In addition to gait parameters, we found more severe motor complications to be associated with deficits also in balance parameters, i.e., sway area. The increased sway may be mediated by disease severity because it occurs at more advanced disease stages as do motor complications [74, 75]. Whereas medication ON states are related to less sway in patients with early PD [87], ON states are associated with more sway in PD subjects with dyskinesia [16, 88], which illustrates the important role motor fluctuations can have on postural control. Associated with this finding, the presence of dyskinesia has been linked to an increased risk of falls [26, 89].

The associations between sway area and clinical parameters on admission shown in this study could perspectively influence clinical decision making in so far as, for example, PD patients with a large sway area and dyskinesia/severe motor fluctuations are offered a targeted therapy or compensation training of these motor complications in the context of PD-MCT to improve postural stability. The identification of shortened steps under motor dual-tasking conditions could lead to targeted motor-motor training considering, e.g., LSVT-BIG, especially in PD patients with motor complications. All this may have a positive impact on functional mobility [90]. In general, we assume that the use of DBP in the context of PD-MCT can help to further individualize therapy [91, 92]. However, further studies with larger samples are undoubtedly needed before this can be implemented in routine clinical treatment. It is also important to ensure that the outcome parameters (and ultimately the therapies) used in the hospital setting are aligned with the maximum possible (individual) relevance to everyday life.

### Changes in device-based and clinical parameters

With PD-MCT, we found an increase in gait velocity, which was pronounced for dual-task conditions, and which exceeded the previously determined minimal clinically important differences [93]. Moreover, stride length increased during motor-motor dual-task walking. It should also be noted that rhythm-related [15] gait parameters did not change significantly.

Additionally, in terms of clinical scores, we found significant improvements in motor severity, balance, dexterity, motor complications, functional mobility, and executive functions. Recent observational studies [18, 19, 21, 22] pointed out similar positive effects of both short-term [18, 19] and long-term [21, 22] PD-MCT. Multidisciplinary inpatient interventions have been shown to improve quality of life, daily functioning and motor symptoms, with differences in effectiveness depending on the duration and intensity of the interventions as well as the stage of the disease [94–99].

Factors potentially contributing to these clinical and objectively measured effects comprise both pharmacological and non-pharmacological components of PD-MCT, specifically increased doses of dopaminergic therapy and physiotherapy [17] or occupational therapy [100]. In more detail, usually 4.5, 3.5, 2.5 and ca. 4 h of physiotherapy, occupational therapy, speech and language therapy, and exercise, respectively, are applied throughout the two-week PD-MCT in our department [19].

On the one hand, the increase in gait velocity might be attributable especially to treadmill and strategy training including cues as well as gait and balance training which are part of both occupational and physiotherapy elements of PD-MCT [18]. For these treatment modalities, a recent meta-analysis has demonstrated moderate to large effects on gait velocity [17]. On the other hand, dopaminergic therapy has also been shown to increase gait velocity [101]. Increased stride length during motor-motor dual-task walking can likewise be attributed to increased LED [101] as well as balance and gait training [17], especially when assuming the application of external cues [101]. Possibly, the amplitude-oriented treatments during PD-MCT, i.e., LSVT-BIG exercises [61] additionally contribute to effects on stride length. Similar favorable effects of inpatient multidisciplinary interventions on gait parameters have been reported in observational [99, 102] and controlled [103] studies. They likewise demonstrated improvements of gait velocity [99, 102, 103] and stride [103] or step [102] length measured by wearable digital devices [102], optoelectronic systems [103], and stopwatches [99] after more [102] or similarly intense [99, 103] interventions with different [102, 103] or similar [99] durations.

Of note, improvements in gait velocity and stride length were marked during dual-task walking, reminding of the baseline finding of shorter strides during dual-task walking in subjects with more severe motor complications. As mentioned above, dual-task conditions are considered more sensitive to subtle deficits [86, 104] which applies especially to motor-motor dual-tasking which has been suggested as a more useful predictor of falls in PD than motor-cognitive dual-tasking [86]. The prominence of these effects during dual-task walking may be additionally explained by the demonstrated improvements in executive functioning, which has albeit not been demonstrated by the correlation analyses discussed below. Evidence suggests that enhanced executive functions can be associated with dopaminergic [105] and exercise-related [106, 107] effects, might contribute to improvements in gait [108] and balance [109], and are, therefore, favorable for daily functioning and quality of life [110]. Interestingly, an observational study [102] showed that changes in supervised step length and in dual-task walking abilities under supervised conditions best-predicted changes in functional mobility after a more intense inpatient multidisciplinary intervention [102]. Crucially, whatever the reasons are for the lack of significant improvement in gait speed and stride length during single-task walking, we argue that the improvement of gait under dual-task conditions is particularly relevant to daily life [85] and may enhance mobility and quality of life.

Importantly, rhythm-related, or ‘qualitative’ gait parameters [43] such as stance, swing, or double support time, did not change significantly after the PD-MCT treatment phase. This may be related to insufficient specificity, intensity, and duration of the intervention or the small sample size of this pilot study. Interestingly, previous studies suggest that dopaminergic therapy could have an effect only on quantitative measures of gait such as velocity but not on measures of gait quality, such as asymmetry or variability [16, 77, 111] and thus improves gait only in part. Individualized training of qualitative gait characteristics may have the potential to enhance daily functioning. The device-based identification of respective deficits (e.g., gait variability) on admission to PD-MCT, along with a clinically-based suspicion that these deficits are relevant to daily life (e.g., history of falls), may prompt tailored training of gait functions during interventions in the future.

### Correlations of changes

Subjects with improvements in motor complications made longer strides at a convenient pace after PD-MCT. These improvements are most likely due to optimization of pharmacological treatment as this improves both motor complications and stride length [16, 101]. Possibly, subjects with high differences in LED between T1 and T2 received more intense occupational or physiotherapy sessions which could also explain longer strides along with weaker motor complications. Interestingly, these associations remind of the baseline correlations between motor complications and stride length, although they occurred under different walking conditions. Conceivably, subjects with motor complications show characteristics predisposing to a parameter-related response to PD-MCT, which should be investigated in future studies.

Surprisingly, a decrease in the severity of motor symptoms was associated with an increase in the sway area during side-by-side stance, although sway did not significantly change throughout the intervention on average. Previous studies suggest an increase in sway with dopaminergic therapy, especially for more advanced PD stages [16]. Therefore, disease severity on MDS-UPDRS III as a predictor of response to PD-MCT [19, 22] may mediate the size-enhancing effects of l-Dopa on postural sway. Importantly, it has been proposed that more sway does not necessarily imply poorer balance performance as movements of the trunk may serve to explore the environment [26, 112].

Finally, an increase in falls efficacy was associated with increases in stance and double support times when performing a cognitive secondary task during walking, albeit neither double support time nor fear of falling changed significantly in the total cohort. Falls efficacy represents self-perceived confidence in performing activities of daily living without falling [113, 114] and is the antonym to fear of falling. That has been shown to be more frequent in PD patients than in healthy age-matched controls [115], is associated with recurrent falls [116], and can be modified by a combination of cognitive-behavioral therapy and exercise [117–119]. Double support time may serve as a measure of dynamic instability [28] and more time with both feet on the ground has been described in more advanced PD stages [69]. PD fallers with high falls efficacy have been observed to turn more quickly, or carelessly than more concerned subjects in the clinical laboratory setting [120], and older adults with high falls efficacy but poor balance have a higher risk for falls than concerned individuals [113]. Thus, the association of increased falls efficacy with increased gait instability during motor-cognitive dual-tasking may be interpreted as a worsening of dynamic equilibrium throughout PD-MCT in subjects who gained confidence in their balance performance (‘confidence-winners’). However, results showed inverted associations for the motor-motor dual-task, implying an improvement of dynamic equilibrium throughout PD-MCT during motor-motor dual-tasking in ‘confidence-winners’. This difference illustrates the importance of having information about environmental conditions and might be explained by more specific training of motor-motor dual-tasks during PD-MCT. As the effects of dopaminergic treatment on motor-motor dual-tasking are limited [111], the improvement in gait stability of ‘confidence-winners’ could be attributed to the effects of gait and balance training during physiotherapy sessions [121]. In any case, the dynamics of falls efficacy seem to be related to dual-task walking abilities, which justifies additional analyses in larger samples.

### Strengths and limitations

Some limitations of this study such as the lack of a control group, the small sample size, and a large number of exploratory comparisons warrant a careful interpretation of the results as changes in clinical scores and device-based parameters, strictly speaking, can only partially be attributed to PD-MCT, or results may have been subjected to the type II error, respectively. The small sample size may have also led to large confidence intervals of the exploratory Spearman correlation coefficients, which warrants a confirmatory breakdown of these results in future analyses. Another point is the supervised assessment condition in the inpatient setting where capacity [10] rather than usual real-life performance [10] is measured due to psychological aspects including social desirability and the Hawthorne effect [92, 102]. A continuous assessment of self-initiated movements in daily life following PD-MCT would have been useful as PD patients walk differently, i.e., slower with shorter strides, in an unsupervised environment [122, 123]. With such data, also more sustained effects of PD-MCT could have been verified or falsified using home-based follow-up assessments. Moreover, continuous assessments could help to enhance the transfer of abilities acquired in the hospital setting to the home setting, which is crucial to sustainably improve daily functioning [92]. Subsequent analyses should include a broader selection of gait parameters comprising data on asymmetry and variability. This study did not include analyses regarding predictors of patient-centered and clinically relevant [91] outcomes of PD-MCT. Knowing factors that strongly influence the therapy response and strengthening them could improve the targeting and efficacy of PD-MCT by taking these predictors into account when selecting patients for PD-MCT or by reinforcing the most effective treatment components.

Of note, this is the first study applying a device-based evaluation in the specific context of the short-term inpatient multidisciplinary PD-MCT concept, which is implemented on a national-level scale in Germany. In addition, we were able to demonstrate associations between clinical and digital parameters in our patients, i.e., a well-defined population with a need for multidisciplinary treatment in a routine hospital setting. Overall, we further promote the use of objective, quantitative data as clinical decision support. For routine clinical care, further research aiming at determining individual target measures is required [27].

## Conclusion

In conclusion, this study confirms associations between device-based and clinical parameters in a PD cohort referred to a PD-MCT and demonstrates the association of shorter stride length with motor complications. Device-based assessment upon admission has the potential to improve the level of individualization of the inpatient therapy. As a result, digital assessment could contribute to more targeted therapy that might ultimately improve the outcomes of such an inpatient therapy approach. We show that PD-MCT improves gait velocity, and stride length during dual-task walking which is a situation that also appears in a real-world setting. Other gait and balance parameters seem to improve less which should be investigated in more detail in future studies. The clinical applicability of these conclusions to clinical practice is limited by the small sample size which warrants replication of the findings in larger studies. Our results suggest motor complications and fear of falling as potential predictors of parameter-related response to PD-MCT, especially regarding dual-task walking. However, the device-based parameters suited best as digital response markers, relevant to clinical and real-world settings, remain to be determined [124]. This study emphasizes the role of gait velocity and stride length as the most promising candidates for digital response markers.

## Supplementary Information

Below is the link to the electronic supplementary material.

Supplementary file1 (DOCX 35 KB)

## Data Availability

The datasets generated during and/or analyzed during the current study are available from the corresponding author on reasonable request.
