# Supplementary material for: Parkinson’s disease multimodal complex treatment improves gait performance: an exploratory wearable digital device-supported study
Source: J Neurol. 2022 Jul 21;269(11):6067–85. doi: 10.1007/s00415-022-11257-x (PMC9553759; doi:10.1007/s00415-022-11257-x)
Supplement: Supplementary file 1 — Supplementary file1 (DOCX 35 KB) [file 415_2022_11257_MOESM1_ESM.docx]

**Table S1** Correlation of MDS-UPDRS IV with corrected and uncorrected step count and stride length for all walking tasks at baseline

| Spearman’s r_s_ | MDS-UPDRS  IV |
| --- | --- |
| **Straight Walk Fast Pace, n=20** |  |
| Step count corrected | 0.54* |
| Step count | 0.60** |
| Stride length corrected | -0.31 |
| Stride length | -0.60** |
| **Straight Walk Normal Pace, n=25** |  |
| Step count corrected | 0.46* |
| Step count | 0.64** |
| Stride length corrected | -0.34 |
| Stride length | -0.64** |
| **Straight Walk Checking Boxes, n=20** |  |
| Step count corrected | 0.49* |
| Step count | 0.76** |
| Stride length corrected | -0.50* |
| Stride length | -0.76** |
| **Straight Walk Subtracting Serial Sevens, n=20** |  |
| Step count corrected | 0.31 |
| Step count | 0.39 |
| Stride length corrected | -0.27 |
| Stride length | -0.39 |
| *p < 0.05 **p < 0.01 |  |

**Table S2** Changes in selected device-based parameters after PD-MCT for the checking boxes walking task

| Variable | T1 | |  | T2 | |  | Δ T2-T1 | |  |  | |
| --- | --- | --- | --- | --- | --- | --- | --- | --- | --- | --- | --- |
|  | M | SD |  | M | SD |  | M | SE |  | BF_10_ | p |
| Straight Walk Checking Boxes, n = 18 |  |  |  |  |  |  |  |  |  |  |  |
| Velocity [m/s] | 1.04 | 0.30 |  | 1.11 | 0.28 |  | 0.07 | 0.02 |  | 12.65 | **0.003*** |
| Step count corrected | 32.3 | 15.6 |  | 22.7 | 8.1 |  | -9.6 | 3.9 |  | 2.30 | **0.023** |
| Step count | 30.7 | 20.6 |  | 21.4 | 8.7 |  | -9.3 | 4.7 |  | 0.96 | 0.066 |
| Stride length corrected [cm] | 82.8 | 37.2 |  | 101.2 | 37.0 |  | 18.4 | 10.2 |  | 0.75 | 0.090 |
| Stride length [cm] | 84.7 | 39.8 |  | 108.3 | 41.3 |  | 23.6 | 10.8 |  | 1.34 | **0.044** |
| *p < 0.01 (Bonferroni-adjusted alpha level, factor of correction (CF): 5) | | | | | | | | | | | |

**Table S3** Correlation of changes in MDS-UPDRS IV and FES-I after PD-MCT with changes in corrected and uncorrected step count and stride length for all walking tasks

| Spearman’s r_s_ | Δ MDS-UPDRS  IV | Δ FES-I |
| --- | --- | --- |
| **Straight Walk Fast Pace, n=20** |  |  |
| Δ Step count corrected | 0.30 | -0.21 |
| Δ Step count | 0.33 | 0.01 |
| Δ Stride length corrected | -0.08 | 0.09 |
| Δ Stride length | -0.44 | 0.04 |
| **Straight Walk Normal Pace, n=25** |  |  |
| Δ Step count corrected | 0.48* | 0.07 |
| Δ Step count | 0.44* | 0.27 |
| Δ Stride length corrected | -0.28 | -0.22 |
| Δ Stride length | -0.40* | -0.36 |
| **Straight Walk Checking Boxes, n=18** |  |  |
| Δ Step count corrected | 0.07 | -0.56* |
| Δ Step count | 0.31 | -0.62** |
| Δ Stride length corrected | -0.33 | 0.45 |
| Δ Stride length | -0.21 | 0.49* |
| **Straight Walk Subtracting Serial Sevens, n=18** |  |  |
| Δ Step count corrected | 0.24 | 0.24 |
| Δ Step count | 0.35 | 0.35 |
| Δ Stride length corrected | -0.05 | -0.46 |
| Δ Stride length | -0.28 | -0.35 |
| *p < 0.05 **p < 0.01 |  |  |
